# Supplementary material for: Toward Accurate RNA Folding Thermodynamics: Evaluation of Enhanced Sampling Methods for Force Field Benchmarking
Source: J Chem Theory Comput. 2026 May 14;22(10):5247–58. doi: 10.1021/acs.jctc.6c00108 (PMC13217565; doi:10.1021/acs.jctc.6c00108)
Supplement: Supplementary file 1 [file ct6c00108_si_001.pdf]

Supporting Information to Toward Accurate RNA Folding Thermodynamics: Evaluation of Enhanced Sampling Methods for Force Field Benchmarking

Petra Kührová,[1,2,#] Vojtěch Mlýnský,[1,#] Ivo Frébort,[2] Jitka Frébortová,[2] Michal Otyepka,[2,3] Jiří Šponer,[1] and Pavel Banáš [2,]*

[1] Institute of Biophysics of the Czech Academy of Sciences, Královopolská 135, 612 00 Brno, Czech Republic
[2] Czech Advanced Technology and Research Institute (CATRIN), Palacký University Olomouc, Šlechtitelů 27, 779 00 Olomouc, Czech Republic
[3] IT4Innovations, VSB – Technical University of Ostrava, 17. listopadu 2172/15, 708 00 Ostrava-Poruba, Czech Republic

Table of Contents

SUPPORTING RESULTS .... - 2 -

SUPPORTING TABLES .... - 6 -

SUPPORTING FIGURES .... - 15 -

REFERENCES .... - 27 -

SUPPORTING RESULTS

UV–vis melting curves and thermodynamic stability. Thermal denaturation of the r(gcGAGAgc) and r(gcGAAAgc) 8-mer RNA hairpins was monitored by UV–vis spectroscopy at 254 nm (see Methods in the main text). For each sequence, melting profiles were measured at two RNA concentrations (5 μM and 10 μM), with two independent measurements performed under each condition. The observed melting curves and derived thermodynamic parameters were concentration independent, consistent with intramolecular hairpin folding.

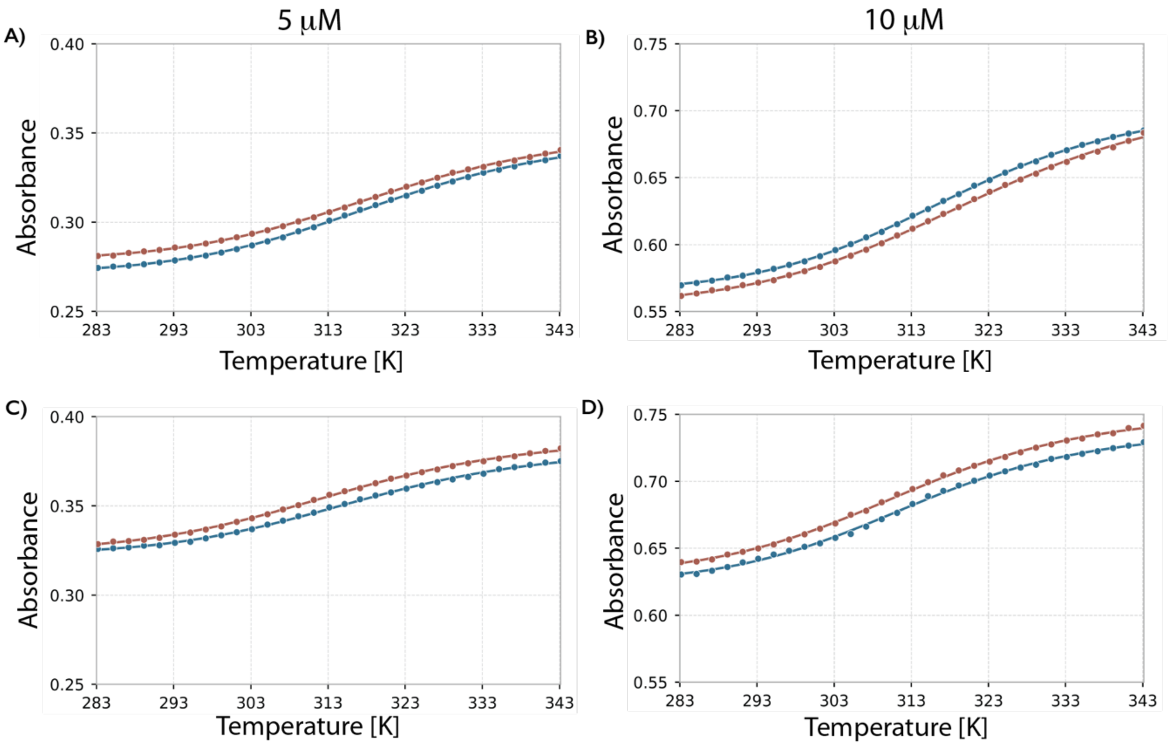

Figure S1: Thermal denaturation profiles of the r(gcGAAAgc) TL (A, B) and r(gcGAGAgc) TL (C, D) obtained by UV–vis spectroscopy. Two independent measurements were performed for each RNA sequence at both concentrations (see Supporting Methods for details).

All melting curves exhibit a single monophasic transition from folded hairpin to unfolded random chain structure and are well described by a two-state model. The fitted thermodynamic parameters ($\Delta H°$, $\Delta S°$, $\Delta G°_{298K}$, and T_m) are summarized in Table S1. The extracted melting temperatures are 314 ± 3 K (40 ± 3 °C) for r(gcGAGAgc) and 319 ± 2 K (46 ± 2 °C) for r(gcGAAAgc), indicating that both sequences form stable hairpin structures under the studied conditions.

Table S1: Thermodynamic parameters for the folding of r(gcGAGAgc) and r(gcGAAAgc) 8-mer TLs.[a]

System	exp. RNA conc. (μM)	$\Delta H°$ (kcal/mol)	$\Delta S°$ (cal/(mol·K))	$\Delta G°_{298K}$ (kcal/mol)	T_m (K) / (°C)	avg. $\Delta G°_{298K}$ (kcal/mol)	avg. T_m (K)
r(gcGAGAgc)	5	-16.05	-50.75	-0.93	316.3 / 43.2	-0.79 ± 0.16	314 ± 3
	5	-15.41	-49.12	-0.77	313.8 / 40.6		
	10	-16.39	-52.44	-0.77	312.6 / 39.4		
	10	-15.64	-50.14	-0.70	311.9 / 38.8		
r(gcGAAAgc)	5	-15.78	-49.41	-1.05	319.3 / 46.2	-1.04 ± 0.04	319 ± 2
	5	-16.00	-50.13	-1.06	319.2 / 46.1		
	10	-16.36	-51.51	-1.02	317.7 / 44.6		
	10	-14.34	-44.73	-1.01	320.6 / 47.5		

[a] $\Delta H°$, $\Delta S°$, and $\Delta G°_{298K}$ are derived from thermal denaturation experiments (two independent measurements at two different RNA concentrations; see Supporting Methods for details) by fitting to a two-state model.

The melting temperature observed for the r(gcGAAAgc) hairpin is somewhat higher than the ~39 °C reported by Hirao et al.[1] The r(gcGAGAgc) tetraloop exhibits a slightly lower melting temperature than r(gcGAAAgc), consistent with the effect of an adenine-to-guanine substitution at the third loop position. This trend is in agreement with previous observations by Leulliot et al. for related GNRA tetraloops in longer (10-mer) hairpin constructs, where substitution at this position modulates thermodynamic stability.[2]

Thermodynamic analysis of simulation data. The folding free energy $\Delta G°_{\text{fold}}$ as reported in the main text was estimated from the simulated folded population (p_{fold}) using a two-state model according to equation presented in the Methods section of the main text, where p_{fold} was defined as the fraction of structures classified as folded based on an εRMSD cutoff of 0.7. The temperature dependence of the folded fraction was further analyzed assuming this two-state equilibrium between folded and unfolded states, yielding the relation:

$$p_{\text{fold}} = \frac{e^{-\frac{\Delta H^\circ_{\text{fold}} - T\Delta S^\circ_{\text{fold}}}{RT}}}{1 + e^{-\frac{\Delta H^\circ_{\text{fold}} - T\Delta S^\circ_{\text{fold}}}{RT}}}$$

While this model provides a convenient approximation, fitting of the folded fraction obtained from the final 7 µs of T-REMD simulations reveals systematic deviations from ideal two-state behavior. In particular, the temperature dependence of p_{fold} cannot be fully captured by a single thermodynamic transition, indicating that the conformational ensemble is more complex. To account for this behavior, we considered a three-state model including folded, unfolded, and misfolded states. In this framework, two equilibrium processes are defined: unfolded → folded ($\Delta G°_{\text{fold}}$) and unfolded → misfolded ($\Delta G°_{\text{misfold}}$), while the equilibrium between folded and misfolded states follows from these relations as a consequence of thermodynamic cycle closure. The resulting expression for the folded fraction is:

$$p_{\text{fold}} = \frac{1}{1 + e^{\frac{\Delta H^\circ_{\text{fold}} - T\Delta S^\circ_{\text{fold}}}{RT}}\left(1 + e^{-\frac{\Delta H^\circ_{\text{misfold}} - T\Delta S^\circ_{\text{misfold}}}{RT}}\right)}$$

This three-state model provides an excellent description of the temperature dependence of the folded fraction observed in the converged T-REMD ensemble (Figure S2). The improved agreement indicates that the definition of folded and unfolded states based on a strict εRMSD cutoff does not fully capture the thermodynamic complexity of the system, with the main limitation arising from the definition of the unfolded ensemble, which cannot be adequately represented as all conformations outside the εRMSD cutoff. In particular, conformations classified as non-folded cannot be treated as a single homogeneous unfolded ensemble.

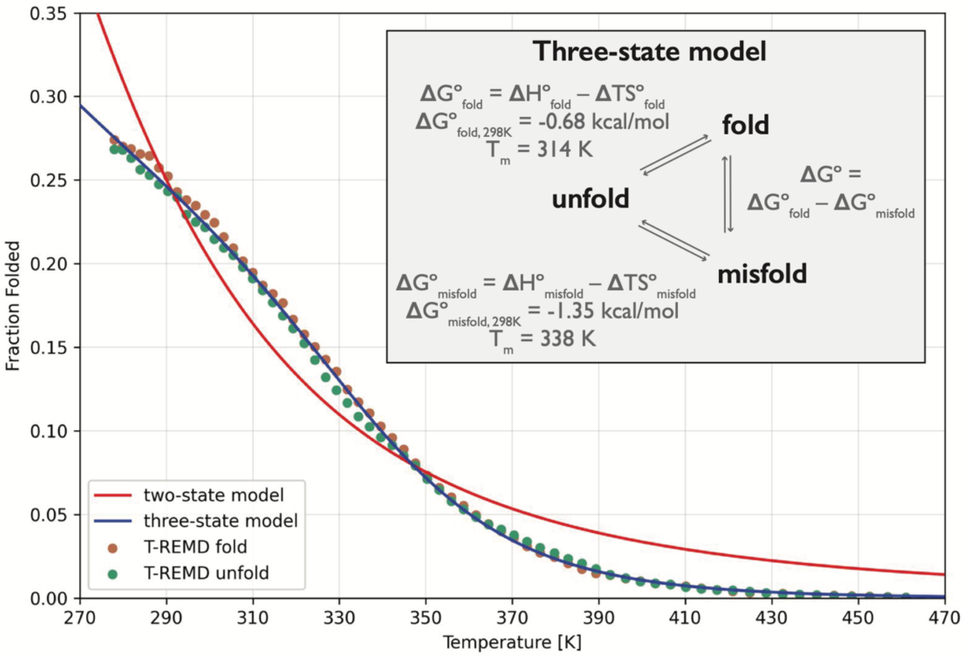

Figure S2: Temperature dependence of the folded population (p_{fold}) obtained from the final 7 µs of 64-replica T-REMD simulations of the GAGA TL. Points correspond to folded fractions from T-REMD simulations initiated from folded (orange) and unfolded (green) ensembles. Solid lines show fits using the two-state (red) and three-state (blue) thermodynamic models. The upper right inset presents the thermodynamic cycle of the three-state model and the corresponding free energy differences at 298 K and associated melting temperatures.

The three-state analysis suggests the presence of a structured misfolded ensemble that is less enthalpically favorable than the native folded state but entropically more favorable, leading to a non-negligible population at intermediate temperatures. The fitted thermodynamic parameters for the unfolded → folded transition are $\Delta H°_{\mathrm{fold}} = -12.97$ kcal/mol and $\Delta S°_{\mathrm{fold}} = -41.3$ cal/(mol·K), corresponding to $\Delta G°_{\mathrm{fold,298K}} = -0.68$ kcal/mol and $T_m \approx 314$ K (41 °C), in very good agreement with experimental values (see Table S1). For the unfolded → misfolded transition, the fitted parameters are $\Delta H°_{\mathrm{misfold}} = -11.41$ kcal/mol and $\Delta S°_{\mathrm{misfold}} = -33.8$ cal/(mol·K), yielding $\Delta G°_{\mathrm{misfold,298K}} = -1.35$ kcal/mol and $T_m \approx 338$ K (65 °C).

The existence of such misfolded states is consistent with the presence of structurally distinct non-native clusters observed in the T-REMD ensemble at lower temperatures (see Figure S3). However, a direct comparison with experiment remains non-trivial, as UV–vis melting experiments report an effective two-state transition based on spectroscopic response. Proper reconciliation of simulation and experiment would therefore require evaluation of the spectroscopic signatures of individual conformational states, including misfolded structures, which is beyond the scope of the present work.

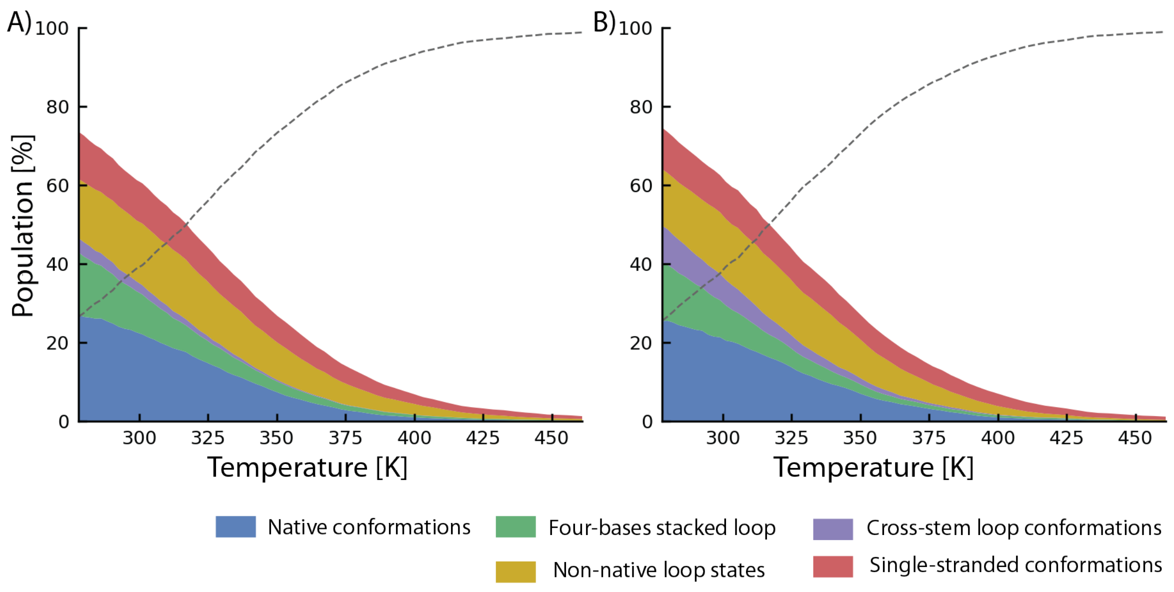

Figure S3: Temperature-dependent populations of conformations in the T-REMD simulations initiated from the folded (A) and unfolded ensemble (B) from final 7 µs. The dashed gray line corresponds to Unassigned states, i.e., frames that cannot be reliably assigned by the clustering approach (algorithm introduced by Rodriguez and Laio[3] in combination with the εRMSD metric[4]; see Ref.[5] for more details). Population of all states are summed to 100%. Numerical values for all states and temperatures are reported in Tables S5 and S6. State definitions are based on εRMSD clustering with the native state defined relative to the experimental hairpin structure (see SI Methods for details).

Definition of conformational states. Conformational states were defined using εRMSD-based clustering. Structures from the T-REMD simulations initiated from the unfolded ensemble (25 µs per replica) were clustered using the εRMSD metric implemented in the Barnaba analysis package, which measures similarity between RNA structures based on the base–base interaction network.[6] Representative structures of the dominant clusters were selected and used as reference conformations for state classification (coordinates are attached as PDB files; *gaga-clusters.zip*).

The reference native structure (folded state) was taken from the 1.04 Å resolution X-ray structure of the sarcin–ricin loop (PDB ID 1Q9A, residues 2658–2663) and extended by a one GC base pair to obtain the r(gcGAGAgc) sequence used in this work. This structure corresponds to the canonical GAGA tetraloop (TL) fold and contains all signature base–base and base–phosphate interactions characteristic of the native state; i.e., (i) the G_{L1}(N2H)···A_{L4}(*pro*-R_P) base-phosphate interaction type 3 (3BPh),[7] which can alternate with G_{L1}(N1H/N2H)···A_{L4}(*pro*-R_P) bifurcated hydrogen-bonds (4BPh interaction), during the simulation, (ii) G_{L1}(N2H)···A_{L4}(N7) base-base, and (iii) G_{L1}(2'-OH)···G_{L3}(N7) sugar-base interaction. The A_{L2}, G_{L3}, and A_{L4} bases form a purine triple base stack and G_{L1} is base-paired with A_{L4} by the trans Hoogsten/Sugar-Edge (tHS)[8] A_{L4}/G_{L1} pattern (see Figure 1 in the main text).

For each simulation frame, εRMSD values relative to these reference structures were calculated using the g-vector representation of RNA conformations. Frames were assigned to the state corresponding to the reference structure with the lowest εRMSD value below the assignment cutoff of 0.7. The following conformational states were considered: the native hairpin conformation ("Native"), conformations with a four-base stacked loop ("4-stack"), cross-stem loop conformations ("Cross"), non-native loop conformations ("Loop"), and single-stranded conformations ("Ss"). The A-form stem conformation ("Stem") was defined using εRMSD evaluated only on the subvector corresponding to the stem residues (residues S-2, S-1, S+1, and S+2; see Figure 1 in the main text). Frames that did not satisfy the assignment criterion were classified as "Unassigned".

These state definitions were used consistently throughout the manuscript, including the temperature-dependent population tables (Tables S5–S10) and the carpet plots shown in Figures 2 and 3 in the main text.

SUPPORTING TABLES

Table S2: Summary of computational characteristics of representative simulations for each enhanced sampling method.[a]

Method	Replicas	Length [μs]	Total sim. time [μs]	Performance [ns/day]	Aggregated GPU time [hours]
REMD	64	25	1,600	617 [b]	62,237
REST2	16	20	320	672 [b]	11,429
	32	20	640	672[b]	22,857
	64	20	1,280	672 [b]	45,714
REHT	20	20	400	399 [c]	24,060
ST-MetaD	12	5	60	669 [b]	2,152
	12	30	360	546 [c]	15,822
	16	5	80	669 [b]	2,870
ST-OPES	12	30	360	480 [d]	18,000

[a] The computational cost is expressed as aggregated GPU time (hours), derived from the total simulation time and measured performance.
[b] NVIDIA A100 40 GB (SXM4).
[c] NVIDIA GeForce RTX 2080 Ti.
[d] NVIDIA GeForce RTX 3080 Ti.

Table S3: Average exchange acceptance rates in REMD, REHT, and REST2 simulations.[a]

Pair	REMD		REHT	REST2		
	unfolded	folded		16 replicas	32 replicas	64 replicas
1↔2	0.26	0.26	0.10	0.61	0.80	0.89
2↔3	0.26	0.26	0.10	0.61	0.80	0.89
3↔4	0.26	0.26	0.17	0.60	0.80	0.89
4↔5	0.26	0.26	0.17	0.59	0.80	0.89
5↔6	0.26	0.25	0.18	0.58	0.79	0.89
6↔7	0.26	0.26	0.10	0.59	0.80	0.89
7↔8	0.26	0.26	0.10	0.58	0.79	0.89
8↔9	0.26	0.26	0.19	0.61	0.79	0.89
9↔10	0.26	0.26	0.19	0.30	0.78	0.89
10↔11	0.26	0.25	0.16	0.32	0.78	0.89
11↔12	0.26	0.25	0.12	0.32	0.79	0.89
12↔13	0.26	0.25	0.11	0.34	0.80	0.89
13↔14	0.26	0.26	0.20	0.35	0.78	0.89
14↔15	0.26	0.25	0.19	0.36	0.78	0.89
15↔16	0.26	0.26	0.21	0.37	0.80	0.89
16↔17	0.26	0.26	0.21		0.80	0.89
17↔18	0.26	0.26	0.21		0.61	0.88
18↔19	0.26	0.25	0.22		0.60	0.88
19↔20	0.26	0.26	0.23		0.62	0.88
20↔21	0.25	0.26			0.61	0.88
21↔22	0.26	0.26			0.63	0.89
22↔23	0.26	0.26			0.63	0.89
23↔24	0.26	0.26			0.63	0.89
24↔25	0.26	0.26			0.63	0.89
25↔26	0.26	0.25			0.64	0.88
26↔27	0.26	0.26			0.63	0.88
27↔28	0.26	0.26			0.65	0.88
28↔29	0.26	0.26			0.64	0.88
29↔30	0.26	0.25			0.66	0.89
30↔31	0.26	0.26			0.65	0.89
31↔32	0.26	0.25			0.66	0.89

32↔33	0.26	0.26		0.89
33↔34	0.26	0.26		0.79
34↔35	0.26	0.26		0.78
35↔36	0.26	0.26		0.79
36↔37	0.26	0.25		0.79
37↔38	0.26	0.26		0.79
38↔39	0.25	0.26		0.79
39↔40	0.26	0.26		0.79
40↔41	0.26	0.26		0.79
41↔42	0.25	0.26		0.80
42↔43	0.26	0.26		0.80
43↔44	0.26	0.26		0.80
44↔45	0.25	0.26		0.79
45↔46	0.25	0.26		0.80
46↔47	0.26	0.26		0.80
47↔48	0.26	0.25		0.80
48↔49	0.25	0.26		0.80
49↔50	0.25	0.26		0.81
50↔51	0.26	0.25		0.80
51↔52	0.25	0.26		0.80
52↔53	0.26	0.25		0.81
53↔54	0.26	0.26		0.81
54↔55	0.26	0.26		0.81
55↔56	0.26	0.25		0.81
56↔57	0.26	0.25		0.81
57↔58	0.26	0.26		0.81
58↔59	0.26	0.26		0.81
59↔60	0.26	0.26		0.81
60↔61	0.25	0.26		0.82
61↔62	0.26	0.25		0.82
62↔63	0.26	0.25		0.81
63↔64	0.26	0.26		0.82

[a] Acceptance rates are reported for each neighboring replica pair as mean values over the entire trajectories. For REMD simulations (starting from both unfolded and folded conformations), exchange probabilities correspond to neighboring replicas in temperature space, whereas for REST2 and REHT simulations, values are averaged over all replica-exchange attempts across all replicas.

Table S4: Average exchange acceptance rates in ST-MetaD and ST-OPES simulations.[a]

Pair				ST-MetaD				ST-OPES
					[b]	[b]	16 replicas	12 replicas
1↔2	0.27	0.26	0.28	0.27	0.19	0.23	0.58	0.29
2↔3	0.26	0.28	0.29	0.30	0.20	0.30	0.61	0.30
3↔4	0.24	0.30	0.31	0.31	0.31	0.15	0.57	0.30
4↔5	0.25	0.29	0.28	0.29	0.31	0.16	0.54	0.16
5↔6	0.27	0.31	0.30	0.33	0.32	0.32	0.52	0.17
6↔7	0.28	0.31	0.32	0.33	0.33	0.33	0.54	0.33
7↔8	0.29	0.33	0.33	0.32	0.25	0.34	0.53	0.34
8↔9	0.34	0.36	0.31	0.32	0.26	0.35	0.56	0.18
9↔10	0.32	0.35	0.35	0.37	0.36	0.36	0.27	0.19
10↔11	0.35	0.36	0.33	0.37	0.28	0.37	0.31	0.37
11↔12	0.34	0.39	0.36	0.39	0.28	0.39	0.33	0.20
12↔13							0.32	
13↔14							0.32	
14↔15							0.34	
15↔16							0.35	

[a] Exchange probabilities are reported for each neighboring replica pair as mean values over all exchange attempts during the simulations. These correspond to instantaneous exchange probabilities reported by the simulation engine, averaged over all recorded exchange steps.
[b] Longer (30 μs) ST-MetaD simulations.

Table S5: Temperature-dependent populations of major states from the final 7 μs of T-REMD simulation initiated from the folded ensemble.[a]

Temp. [K]	Native	4-stack	Cross	Loop	Ss	Stem	Unassigned
278.0	27.0	18.4	3.4	11.9	8.0	1.5	30.0
279.9	26.4	17.8	3.4	12.0	7.8	1.5	31.0
281.9	26.3	17.2	3.3	12.1	7.5	1.5	32.2
284.0	26.0	16.3	3.1	12.2	7.4	1.6	33.4
286.1	26.0	15.7	2.9	12.2	7.3	1.6	34.2
288.3	25.3	15.1	2.8	12.3	7.3	1.6	35.6
290.4	24.7	14.5	2.8	12.3	7.2	1.6	36.9
292.5	23.9	13.7	2.5	12.4	7.1	1.7	38.7
294.7	23.4	13.2	2.5	12.1	6.9	1.7	40.1
296.8	23.1	12.7	2.4	12.0	6.8	1.7	41.3
**299.0**	22.5	12.3	2.3	11.9	6.8	1.8	42.4
301.1	22.0	11.9	2.2	12.0	6.8	1.9	43.2
303.3	21.2	11.3	2.1	12.1	6.8	1.9	44.5
305.5	20.6	10.7	2.0	11.9	6.7	2.0	46.1
307.7	19.9	10.2	1.9	11.8	6.8	2.0	47.5
310.0	19.2	9.8	1.8	12.0	6.7	2.0	48.6
312.3	18.5	9.1	1.7	12.0	6.5	2.1	50.2
314.6	18.0	8.7	1.6	11.9	6.5	2.1	51.3
317.0	17.5	8.2	1.4	11.6	6.2	2.2	53.0

319.3	16.5	7.8	1.3	11.1	6.1	2.3	54.8
321.9	15.6	7.3	1.2	11.0	6.1	2.2	56.6
324.4	14.8	7.0	1.1	10.7	5.9	2.2	58.3
326.8	14.1	6.6	1.0	10.3	5.9	2.3	59.9
329.3	13.4	6.2	0.9	9.5	5.7	2.2	62.1
331.9	12.3	6.0	0.8	9.4	5.5	2.3	63.7
334.4	11.6	5.5	0.8	9.2	5.2	2.3	65.4
337.0	11.0	5.2	0.7	8.8	5.2	2.4	66.8
339.6	10.2	4.8	0.6	8.1	5.1	2.5	68.7
342.2	9.4	4.4	0.5	7.7	4.9	2.3	70.7
344.9	8.8	4.1	0.5	7.4	4.7	2.3	72.2
347.6	7.9	3.8	0.4	7.0	4.6	2.3	73.9
350.3	7.2	3.6	0.3	6.7	4.5	2.3	75.4
353.1	6.4	3.4	0.3	6.4	4.4	2.2	76.8
355.9	5.9	3.1	0.3	6.0	4.3	2.1	78.4
358.7	5.4	2.8	0.2	5.5	4.1	2.0	79.9
361.6	4.8	2.6	0.2	5.2	3.8	2.0	81.4
364.5	4.3	2.4	0.2	4.7	3.7	1.9	82.9
367.4	3.9	2.2	0.1	4.5	3.5	1.8	84.0
370.4	3.4	1.9	0.1	4.1	3.3	1.7	85.5
373.4	2.9	1.7	0.1	3.8	3.1	1.6	86.8
376.4	2.6	1.5	0.1	3.5	2.9	1.5	87.9
379.7	2.3	1.4	0.1	3.1	2.8	1.5	88.9
382.9	1.9	1.3	0.1	2.8	2.5	1.3	90.0
386.1	1.6	1.2	0.1	2.5	2.3	1.2	91.1
389.3	1.4	1.0	0.1	2.3	2.1	1.1	91.9
392.6	1.3	0.8	0.1	2.2	1.9	1.0	92.6
396.2	1.1	0.8	0.1	2.0	1.7	0.9	93.4
399.6	0.9	0.7	0.0	1.9	1.5	0.9	94.1
403.1	0.8	0.6	0.0	1.6	1.4	0.8	94.8
406.6	0.7	0.5	0.1	1.4	1.3	0.8	95.2
410.1	0.6	0.4	0.0	1.2	1.2	0.8	95.7
413.7	0.5	0.4	0.0	1.1	1.1	0.7	96.2
417.3	0.4	0.3	0.0	0.9	1.0	0.6	96.7
421.0	0.4	0.3	0.0	0.8	0.9	0.6	97.0
424.9	0.3	0.3	0.0	0.7	0.9	0.5	97.3
428.7	0.3	0.3	0.0	0.5	0.8	0.5	97.5
432.5	0.3	0.2	0.0	0.4	0.8	0.5	97.8
436.4	0.2	0.2	0.0	0.4	0.7	0.4	98.0
440.4	0.2	0.2	0.0	0.3	0.7	0.4	98.3
444.4	0.1	0.1	0.0	0.3	0.6	0.4	98.4
448.5	0.1	0.1	0.0	0.3	0.5	0.3	98.6
452.6	0.1	0.1	0.0	0.2	0.5	0.3	98.8

456.8	0.1	0.1	0.0	0.2	0.4	0.3	98.9
461.0	0.0	0.1	0.0	0.2	0.4	0.2	99.1

[a] For each temperature, the table reports the percentage of frames in native ("Native"), A-form stem ("Stem"), four-base stacked loop ("4-stack"), cross-stem loop ("Cross"), non-native loop ("Loop"), single-stranded ("SS"), and unassigned ("Unassigned") conformations. State definitions are based on εRMSD clustering, with the native state defined relative to the experimental hairpin structure (see Supporting Methods). The highlighted temperature (in bold) indicates the reference temperature used in subsequent analyses.

Table S6: Temperature-dependent populations of major states from the final 7 μs of T-REMD simulation initiated from the unfolded ensemble.[a]

Temperature [K]	Native	4-stack	Cross	Loop	Ss	Stem	Unassigned
278.0	25.4	16.2	8.8	10.3	7.4	0.6	31.2
279.9	25.5	15.6	8.5	10.3	7.2	0.6	32.2
281.9	25.0	15.1	8.0	10.5	7.1	0.7	33.4
284.0	24.4	14.4	7.9	10.5	7.2	0.6	35.0
286.1	24.0	14.0	7.5	10.6	7.1	0.7	36.1
288.3	23.5	13.2	7.2	11.1	7.0	0.8	37.1
290.4	23.1	12.5	6.9	11.1	7.0	0.8	38.5
292.5	22.9	12.0	6.5	11.1	7.0	0.8	39.7
294.7	21.9	11.7	6.4	11.2	6.9	0.8	41.0
296.8	21.5	11.0	6.4	11.3	6.9	0.9	42.0
**299.0**	21.3	10.3	6.1	11.3	6.8	0.9	43.3
301.1	20.4	9.9	5.8	11.2	6.8	0.9	45.0
303.3	20.1	9.1	5.6	11.1	6.9	0.9	46.2
305.5	19.6	8.8	5.3	11.2	6.9	1.0	47.1
307.7	18.9	8.5	4.9	11.1	6.9	1.0	48.6
310.0	18.1	8.0	4.7	11.0	6.6	1.1	50.5
312.3	17.5	7.6	4.3	11.2	6.6	1.1	51.7
314.6	16.8	7.1	3.9	10.8	6.3	1.1	53.9
317.0	16.1	6.7	3.7	10.7	6.2	1.2	55.4
319.3	15.5	6.5	3.4	10.5	6.1	1.2	56.9
321.9	14.7	6.1	3.1	10.2	5.9	1.2	58.8
324.4	13.8	5.7	2.9	10.0	5.9	1.3	60.4
326.8	12.9	5.3	2.7	9.7	5.9	1.3	62.2
329.3	12.0	4.9	2.5	9.5	5.7	1.5	63.8
331.9	11.4	4.7	2.3	9.4	5.7	1.5	65.0
334.4	10.6	4.5	2.1	9.2	5.6	1.5	66.5
337.0	10.0	4.2	2.0	8.8	5.6	1.5	67.9
339.6	9.3	3.9	1.9	8.5	5.5	1.5	69.5
342.2	8.9	3.4	1.6	7.9	5.3	1.5	71.3
344.9	8.3	3.2	1.6	7.6	5.1	1.4	72.8
347.6	7.6	2.9	1.5	7.2	4.8	1.5	74.6
350.3	6.8	2.7	1.3	6.7	4.6	1.5	76.2
353.1	6.2	2.5	1.1	6.2	4.5	1.6	78.0

355.9	5.5	2.3	1.1	5.7	4.4	1.4	79.7
358.7	5.1	2.1	1.0	5.4	4.1	1.4	80.9
361.6	4.7	1.8	0.9	4.9	4.0	1.3	82.4
364.5	4.3	1.6	0.7	4.6	4.0	1.3	83.5
367.4	4.0	1.5	0.6	4.2	3.8	1.3	84.6
370.4	3.6	1.4	0.6	4.0	3.6	1.3	85.6
373.4	3.2	1.2	0.5	3.7	3.4	1.2	86.8
376.4	2.9	1.1	0.4	3.2	3.3	1.1	87.9
379.7	2.6	1.1	0.4	3.0	3.2	1.0	88.7
382.9	2.2	1.0	0.4	2.6	3.0	0.9	89.9
386.1	2.0	0.9	0.3	2.4	2.8	0.8	90.9
389.3	1.7	0.8	0.2	2.2	2.6	0.8	91.8
392.6	1.3	0.7	0.2	2.0	2.5	0.7	92.6
396.2	1.1	0.6	0.1	1.7	2.3	0.7	93.4
399.6	0.9	0.5	0.1	1.5	2.2	0.6	94.2
403.1	0.8	0.5	0.1	1.2	2.0	0.6	94.8
406.6	0.7	0.5	0.1	1.0	1.8	0.5	95.4
410.1	0.6	0.4	0.1	0.8	1.6	0.5	96.0
413.7	0.5	0.4	0.1	0.7	1.4	0.5	96.4
417.3	0.4	0.4	0.1	0.6	1.3	0.4	96.9
421.0	0.4	0.3	0.1	0.5	1.2	0.4	97.1
424.9	0.4	0.3	0.0	0.5	1.1	0.3	97.5
428.7	0.3	0.3	0.0	0.4	0.9	0.3	97.8
432.5	0.2	0.2	0.0	0.3	0.8	0.2	98.2
436.4	0.2	0.1	0.0	0.3	0.7	0.2	98.5
440.4	0.2	0.1	0.0	0.3	0.6	0.2	98.6
444.4	0.1	0.1	0.0	0.2	0.6	0.2	98.7
448.5	0.1	0.1	0.0	0.2	0.6	0.2	98.9
452.6	0.1	0.1	0.0	0.2	0.5	0.2	99.0
456.8	0.1	0.1	0.0	0.1	0.5	0.1	99.1
461.0	0.0	0.1	0.0	0.1	0.4	0.1	99.2

[a] See Table S5 footnote for details.

Table S7: Temperature-dependent populations of major states from the final 7 μs of REST2 simulation with 16 replicas.[a]

Temperature [K]	Native	4-stack	Cross	Loop	Ss	Stem	Unassigned
273.3	28.0	23.9	0.0	2.1	5.5	0.6	39.9
279.1	26.8	20.2	0.0	2.0	5.0	0.9	45.2
285.2	24.2	16.9	0.0	2.0	5.5	0.9	50.4
291.5	23.1	14.1	0.0	2.1	5.8	0.9	54.0
**298.2**	19.6	13.3	0.0	2.2	6.6	1.0	57.3
305.4	16.4	11.1	0.0	2.2	6.3	1.2	62.8
312.3	13.5	9.2	0.0	2.2	6.1	1.4	67.5
319.9	11.3	6.9	0.0	2.5	5.7	1.3	72.2
327.2	8.7	5.9	0.0	1.9	6.0	1.2	76.2
342.8	4.6	3.9	0.0	1.3	4.6	1.1	84.5

359.1	2.2	2.4	0.0	1.0	3.1	0.7	90.7
376.1	1.2	1.5	0.0	0.8	2.1	0.6	93.9
394.0	0.5	0.6	0.0	0.3	1.3	0.3	97.0
412.8	0.2	0.3	0.0	0.2	1.0	0.1	98.2
432.4	0.0	0.2	0.0	0.0	0.4	0.1	99.2
453.0	0.0	0.1	0.0	0.0	0.3	0.0	99.5

[a] See Table S5 footnote for details.

Table S8: Temperature-dependent populations of major states from the final 7 μs of REST2 simulation with 32 replicas.[a]

Temperature [K]	Native	4-stack	Cross	Loop	Ss	Stem	Unassigned
273.3	19.6	18.0	0.0	11.1	8.7	2.9	39.8
276.2	19.8	16.8	0.0	11.5	9.0	2.7	40.1
279.1	19.6	15.4	0.0	10.6	8.9	3.0	42.4
282.1	18.8	15.4	0.0	10.7	9.5	3.0	42.6
285.2	17.8	14.3	0.0	11.7	9.1	2.9	44.2
288.3	17.0	13.6	0.0	11.1	9.0	2.5	46.7
291.5	16.8	13.1	0.0	10.9	8.6	2.6	48.0
294.8	15.6	12.6	0.0	10.8	9.1	2.3	49.6
**298.2**	14.4	11.8	0.0	10.4	9.7	2.5	51.2
301.7	14.1	11.0	0.0	9.5	9.8	2.4	53.3
305.4	12.6	10.8	0.0	9.6	9.0	2.6	55.5
308.8	11.6	9.5	0.0	9.3	8.3	2.4	58.9
312.3	10.6	8.8	0.0	8.7	8.9	2.0	61.0
316.1	9.5	7.8	0.0	8.4	8.9	1.7	63.7
319.9	8.5	7.8	0.0	7.5	8.2	1.7	66.3
323.5	7.9	7.1	0.0	7.3	8.7	1.8	67.2
327.2	6.8	6.2	0.0	6.5	7.5	1.7	71.2
334.8	5.3	5.0	0.0	5.4	6.5	1.5	76.3
342.8	3.7	4.5	0.0	4.7	6.0	1.1	79.9
350.7	2.8	3.4	0.0	3.5	5.0	1.0	84.3
359.1	1.9	2.4	0.0	2.7	4.0	0.7	88.4
367.4	1.2	1.9	0.0	2.4	3.2	0.8	90.4
376.1	1.1	1.6	0.0	1.7	2.7	0.4	92.6
384.9	0.4	1.0	0.0	1.3	2.1	0.2	95.0
394.0	0.3	0.6	0.0	0.6	1.7	0.3	96.5
403.2	0.3	0.7	0.0	0.5	1.3	0.2	97.1
412.8	0.1	0.4	0.0	0.4	0.9	0.2	98.0
422.3	0.1	0.3	0.0	0.3	0.6	0.1	98.7
432.4	0.0	0.2	0.0	0.2	0.4	0.1	99.2
442.4	0.0	0.2	0.0	0.1	0.4	0.2	99.1
453.0	0.0	0.2	0.0	0.0	0.3	0.1	99.4
463.5	0.0	0.1	0.0	0.0	0.2	0.1	99.6

[a] See Table S5 footnote for details.

Table S9: Temperature-dependent populations of major states from the final 7 μs of REST2 simulation with 64 replicas.[a]

Temperature [K]	Native	4-stack	Cross	Loop	Ss	Stem	Unassigned
273.3	13.5	31.0	1.3	11.8	6.5	0.1	35.8
274.8	12.8	30.3	1.0	12.4	6.8	0.1	36.6
276.2	13.0	29.3	1.2	12.3	6.8	0.1	37.4
277.7	13.9	27.7	1.2	12.1	6.6	0.1	38.2
279.1	12.9	27.3	1.1	11.8	7.1	0.2	39.6
280.6	12.2	27.4	1.0	12.1	7.1	0.2	40.0
282.1	13.2	27.3	1.1	11.7	7.6	0.1	39.1
283.7	11.6	26.2	0.9	11.6	7.9	0.2	41.6
285.2	12.1	25.5	0.8	12.0	7.6	0.1	41.9

286.8	12.0	24.6	0.8	11.0	8.1	0.2	43.4
288.3	11.7	23.9	0.6	11.5	7.9	0.2	44.3
289.9	11.7	23.9	0.6	10.6	8.2	0.2	44.8
291.5	10.2	22.5	0.8	11.3	7.9	0.2	47.0
293.2	10.6	22.4	0.7	11.2	7.7	0.2	47.2
294.8	11.2	22.2	0.7	10.3	8.5	0.2	46.9
296.5	10.2	20.6	0.6	10.9	8.3	0.1	49.2
**298.2**	10.3	19.4	0.5	10.4	8.7	0.2	50.5
299.9	9.8	18.5	0.6	10.4	8.5	0.3	51.9
301.7	9.5	19.0	0.6	9.7	8.2	0.2	53.0
303.6	8.8	18.3	0.5	9.4	9.1	0.2	53.8
305.4	8.7	16.8	0.4	9.5	8.7	0.2	55.6
307.1	8.6	16.6	0.4	8.7	8.7	0.3	56.7
308.8	8.6	15.5	0.3	8.0	9.0	0.2	58.3
310.6	7.9	15.2	0.3	8.3	9.6	0.3	58.5
312.3	7.6	14.4	0.4	8.3	9.0	0.2	60.0
314.2	7.6	13.9	0.3	7.8	8.7	0.3	61.4
316.1	7.3	13.7	0.2	7.8	9.0	0.3	61.7
318.0	6.9	13.2	0.3	7.0	8.5	0.2	63.8
319.9	6.4	12.3	0.3	7.0	9.0	0.1	64.9
321.7	5.9	11.9	0.2	6.5	9.2	0.3	65.9
323.5	6.0	10.9	0.2	6.5	9.6	0.3	66.5
325.3	5.6	10.6	0.2	5.9	8.5	0.4	68.9
327.2	5.4	10.2	0.2	5.9	8.4	0.3	69.6
331.0	4.5	9.2	0.2	5.5	8.0	0.3	72.3
334.8	3.8	7.8	0.2	4.1	7.9	0.3	76.0
338.7	3.3	7.5	0.0	3.9	7.1	0.3	77.9
342.8	3.2	6.2	0.1	3.2	7.8	0.2	79.3
346.7	2.3	5.2	0.1	3.3	6.7	0.3	82.2
350.7	2.2	4.7	0.0	2.8	6.6	0.2	83.5
354.8	1.8	3.8	0.0	2.2	6.1	0.2	85.8
359.1	1.7	3.5	0.0	2.4	5.4	0.2	86.8
363.2	1.3	2.7	0.0	1.8	5.1	0.2	88.9
367.4	1.0	2.5	0.0	1.7	4.9	0.2	89.6
371.7	0.9	1.9	0.0	1.4	4.0	0.2	91.6
376.1	0.6	1.9	0.0	1.0	4.1	0.1	92.3
380.5	0.6	1.6	0.0	0.6	3.6	0.2	93.4
384.9	0.5	1.3	0.0	0.6	3.0	0.2	94.4
389.4	0.2	1.1	0.0	0.6	2.7	0.1	95.3
394.0	0.2	0.7	0.0	0.4	2.7	0.1	95.9
398.5	0.1	0.7	0.0	0.4	2.5	0.1	96.3
403.2	0.1	0.6	0.0	0.3	1.9	0.1	96.9
407.9	0.1	0.4	0.0	0.3	1.6	0.0	97.6
412.8	0.1	0.4	0.0	0.2	1.5	0.1	97.8
417.5	0.1	0.3	0.0	0.2	1.4	0.1	98.0
422.3	0.0	0.3	0.0	0.1	1.1	0.1	98.4
427.3	0.0	0.2	0.0	0.1	0.9	0.1	98.6
432.4	0.1	0.2	0.0	0.1	0.8	0.1	98.8
437.4	0.0	0.2	0.0	0.1	0.7	0.0	99.0
442.4	0.0	0.1	0.0	0.1	0.6	0.1	99.1
447.6	0.0	0.1	0.0	0.0	0.5	0.0	99.3
453.0	0.0	0.1	0.0	0.0	0.4	0.0	99.4
458.2	0.0	0.1	0.0	0.0	0.4	0.0	99.5
463.5	0.0	0.0	0.0	0.0	0.4	0.0	99.6
468.9	0.0	0.1	0.0	0.1	0.3	0.0	99.6

[a] See Table S5 footnote for details.

Table S10: Temperature-dependent populations of major states from the final 7 μs of REHT simulations with 20 replicas.[a]

Temperature [K] Solvent	Solute	Native	4-stack	Cross	Loop	Ss	Stem	Unassigned
293.5	281.6	25.5	14.7	0.0	17.8	9.6	0.2	32.2
295.7	289.5	26.1	14.9	0.0	15.5	10.7	0.3	32.6
298.0	**298.0**	24.5	14.5	0.0	13.3	11.1	0.3	36.3
300.3	307.1	23.5	12.9	0.0	11.3	10.7	0.3	41.2
302.7	316.5	18.2	9.8	0.0	9.4	10.5	0.6	51.5
305.0	326.2	14.4	8.4	0.0	7.2	9.5	0.7	59.9
307.4	336.1	8.5	6.5	0.0	5.3	8.3	0.7	70.8
309.8	346.4	6.0	4.3	0.0	3.2	6.3	0.6	79.6
312.2	357.0	3.0	2.8	0.0	2.1	4.4	0.5	87.2
314.6	367.9	1.6	1.7	0.0	1.2	3.5	0.4	91.5
317.1	379.1	0.8	0.8	0.0	0.7	2.2	0.2	95.3
319.6	390.7	0.4	0.6	0.0	0.3	1.8	0.2	96.7
322.0	402.6	0.2	0.2	0.0	0.2	1.1	0.3	98.0
324.5	414.9	0.1	0.2	0.0	0.1	0.9	0.1	98.6
327.1	427.6	0.0	0.1	0.0	0.1	0.3	0.1	99.5
329.6	440.6	0.0	0.1	0.0	0.0	0.2	0.0	99.6
332.2	454.1	0.0	0.0	0.0	0.0	0.3	0.0	99.7
334.8	468.0	0.0	0.0	0.0	0.0	0.1	0.0	99.8
337.4	482.3	0.0	0.0	0.0	0.0	0.1	0.0	99.9
340.0	497.0	0.0	0.0	0.0	0.0	0.1	0.0	99.9

[a] See Table S5 footnote for details.

SUPPORTING FIGURES

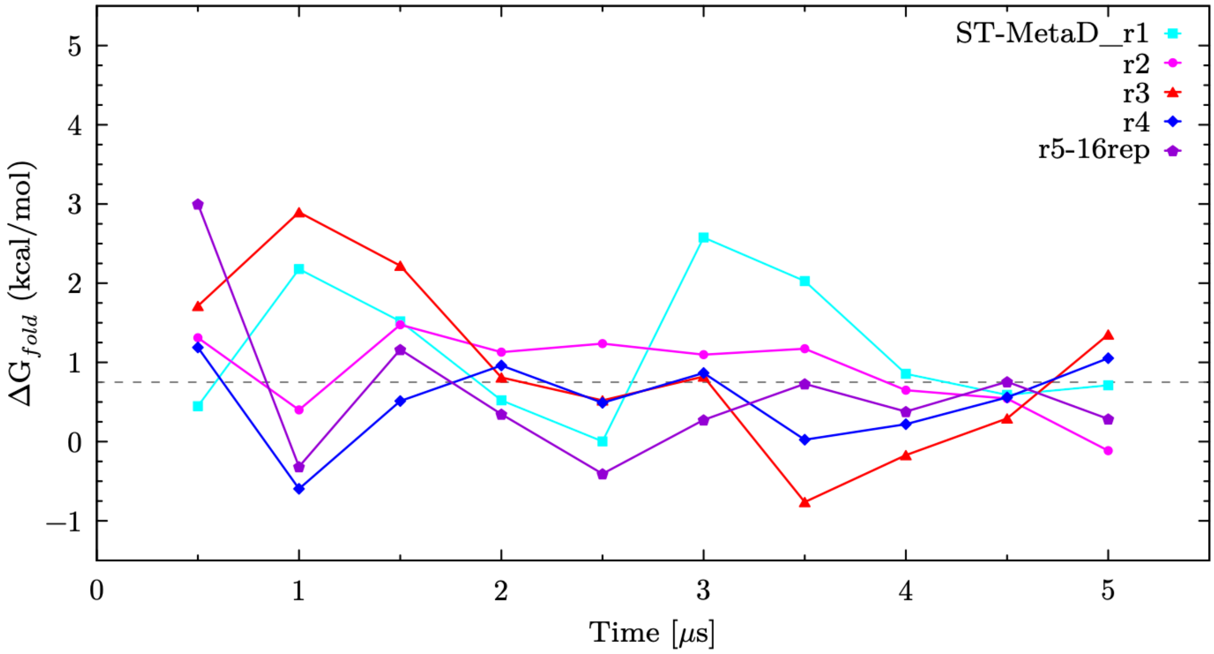

Figure S4: Time evolution of folding free energies (ΔG°$_{fold}$) from five 5 μs-long ST-MetaD simulations estimated from the corresponding reweighted ensembles using standard εRMSD (see Methods in the main text), with each line representing values computed using the instantaneous bias accumulated up to the given time.

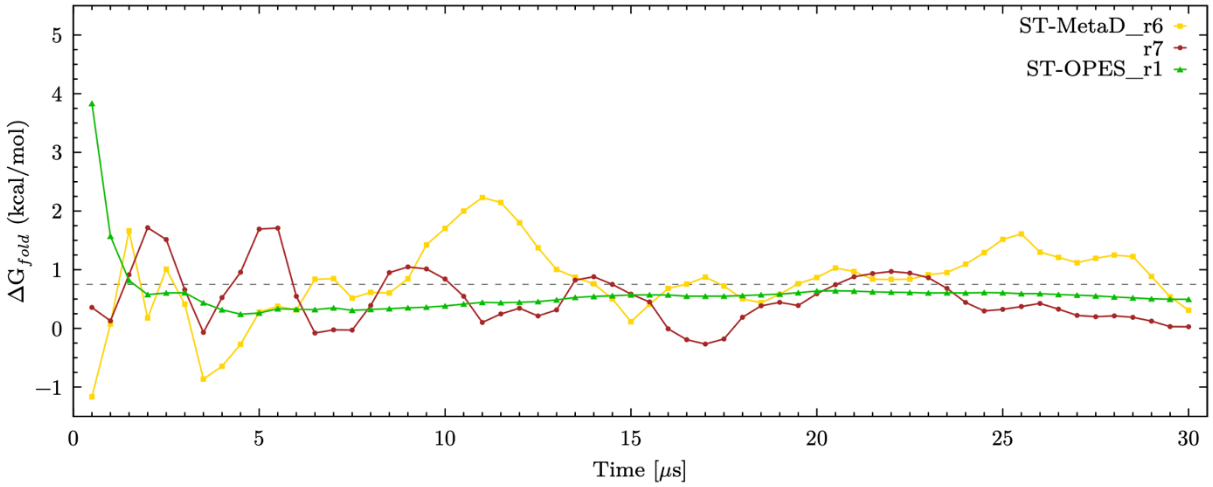

Figure S5: Time evolution of folding free energies (ΔG°$_{fold}$) from two 30 μs-long ST-MetaD simulations and one 30 μs-long ST-OPES simulation estimated from the corresponding reweighted ensembles using standard εRMSD (see Methods in the main text), with each line representing values computed using the instantaneous bias accumulated up to the given time.

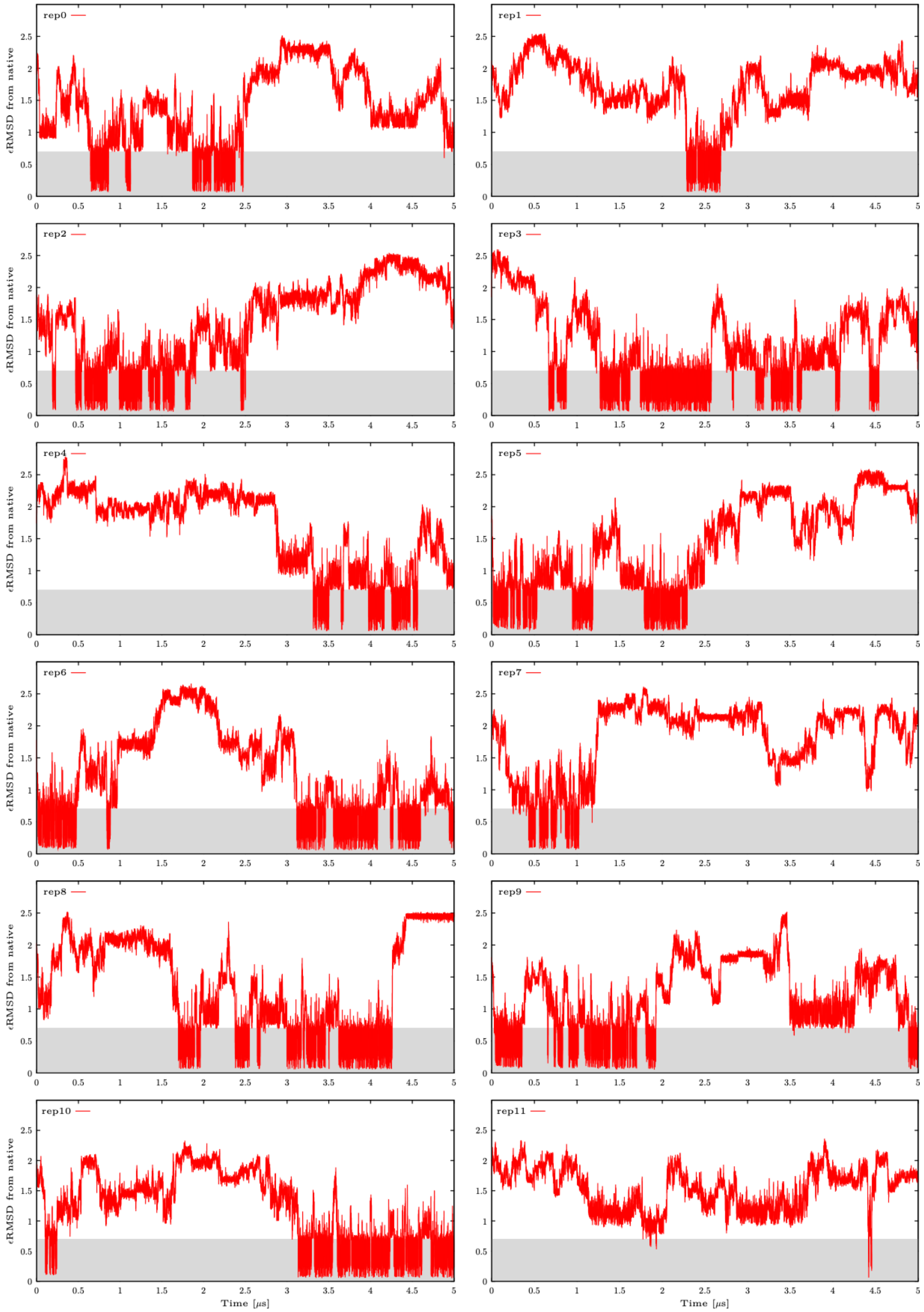

Figure S6: Calculated εRMSD relative to the native state for all twelve continuous (demultiplexed) replicas from the first ST-MetaD simulation (5 µs-long) of the 8-mer GAGA TL. εRMSD values were computed every 50 ps. The shaded region highlights structures close to the native state (εRMSD < 0.7).

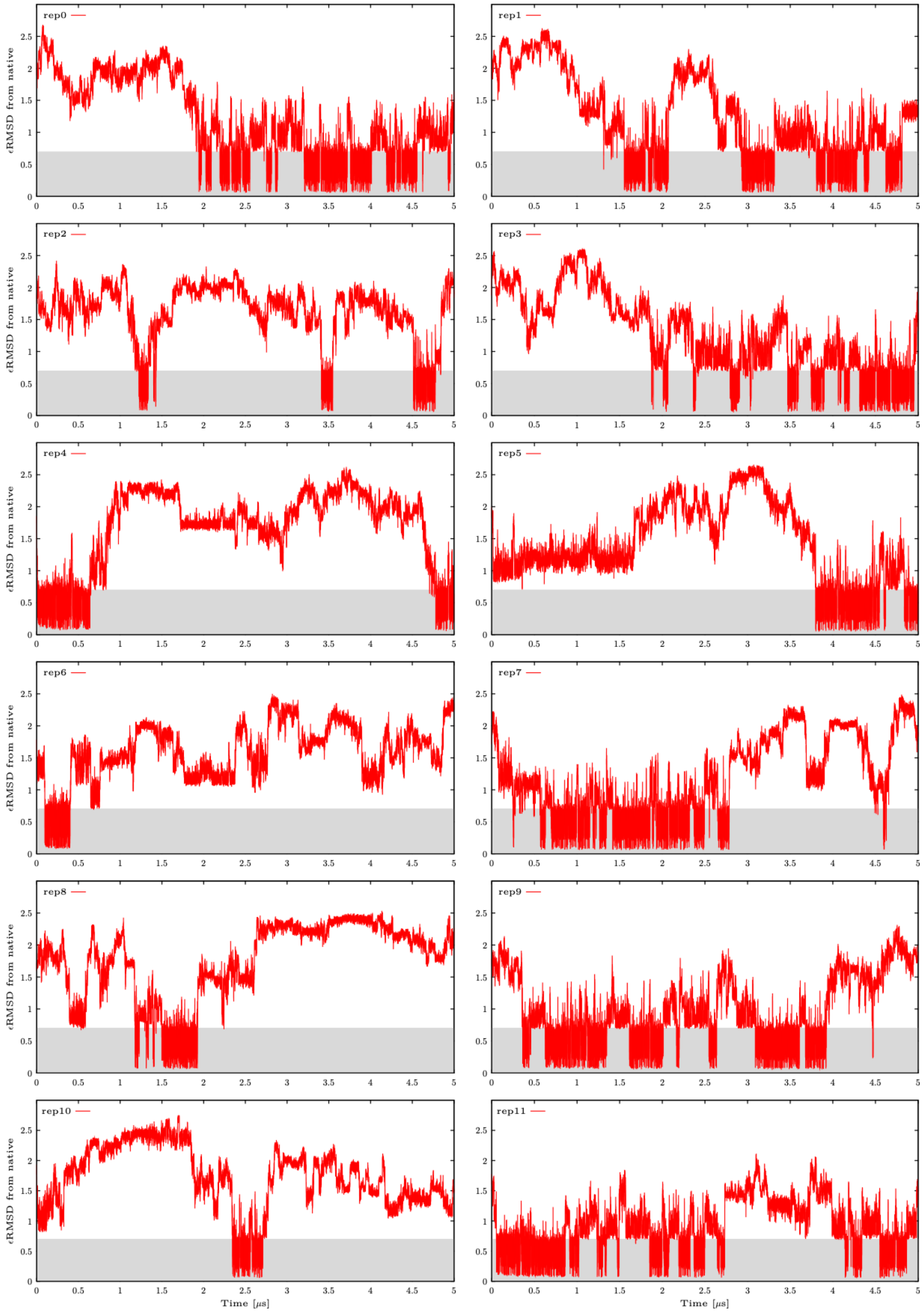

Figure S7: Calculated εRMSD relative to the native state for all twelve continuous (demultiplexed) replicas from the second ST-MetaD simulation (5 µs-long) of the 8-mer GAGA TL. See Figure S6 for more details.

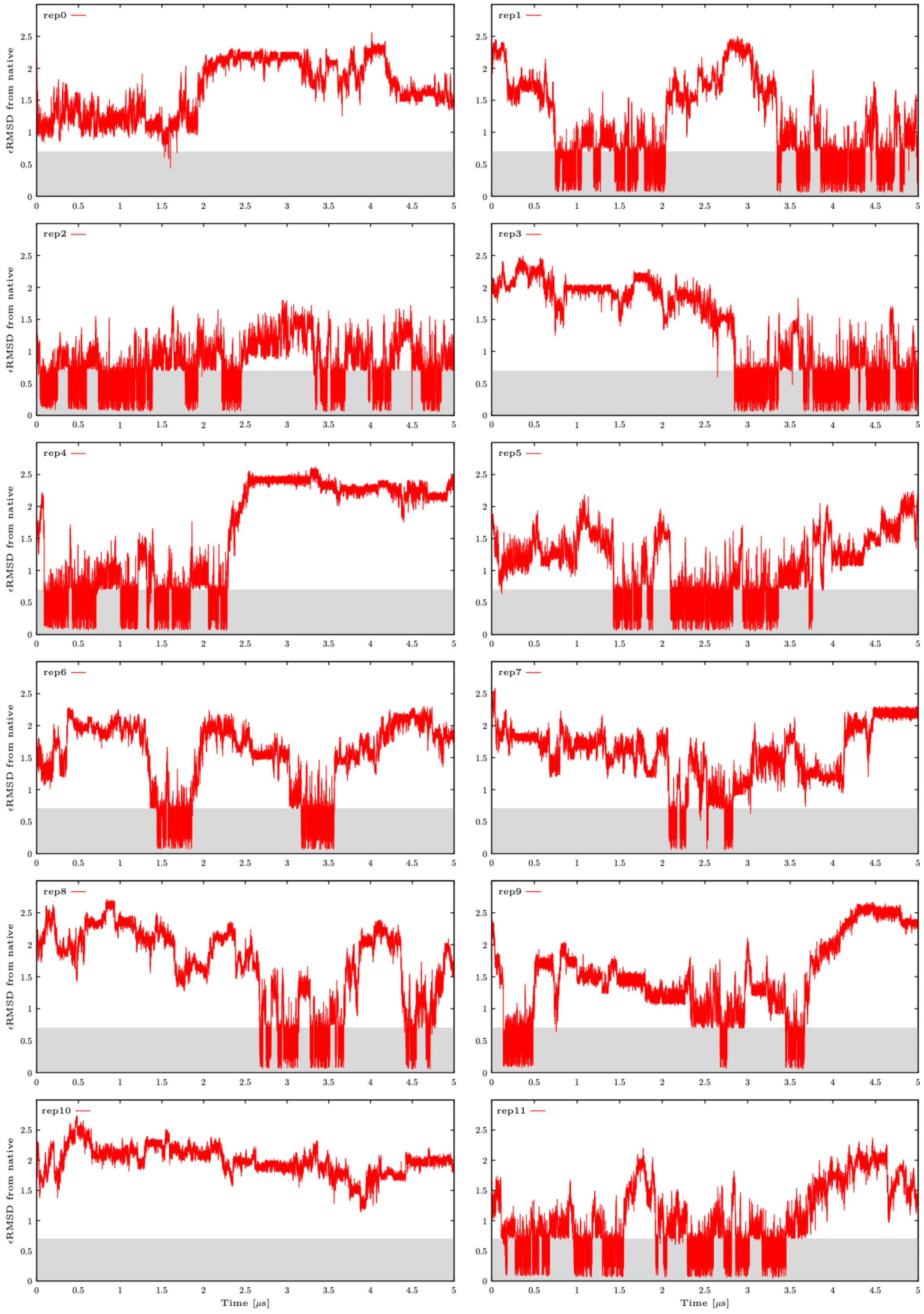

Figure S8: Calculated εRMSD relative to the native state for all twelve continuous (demultiplexed) replicas from the third ST-MetaD simulation (5 μs-long) of the 8-mer GAGA TL. See Figure S6 for more details.

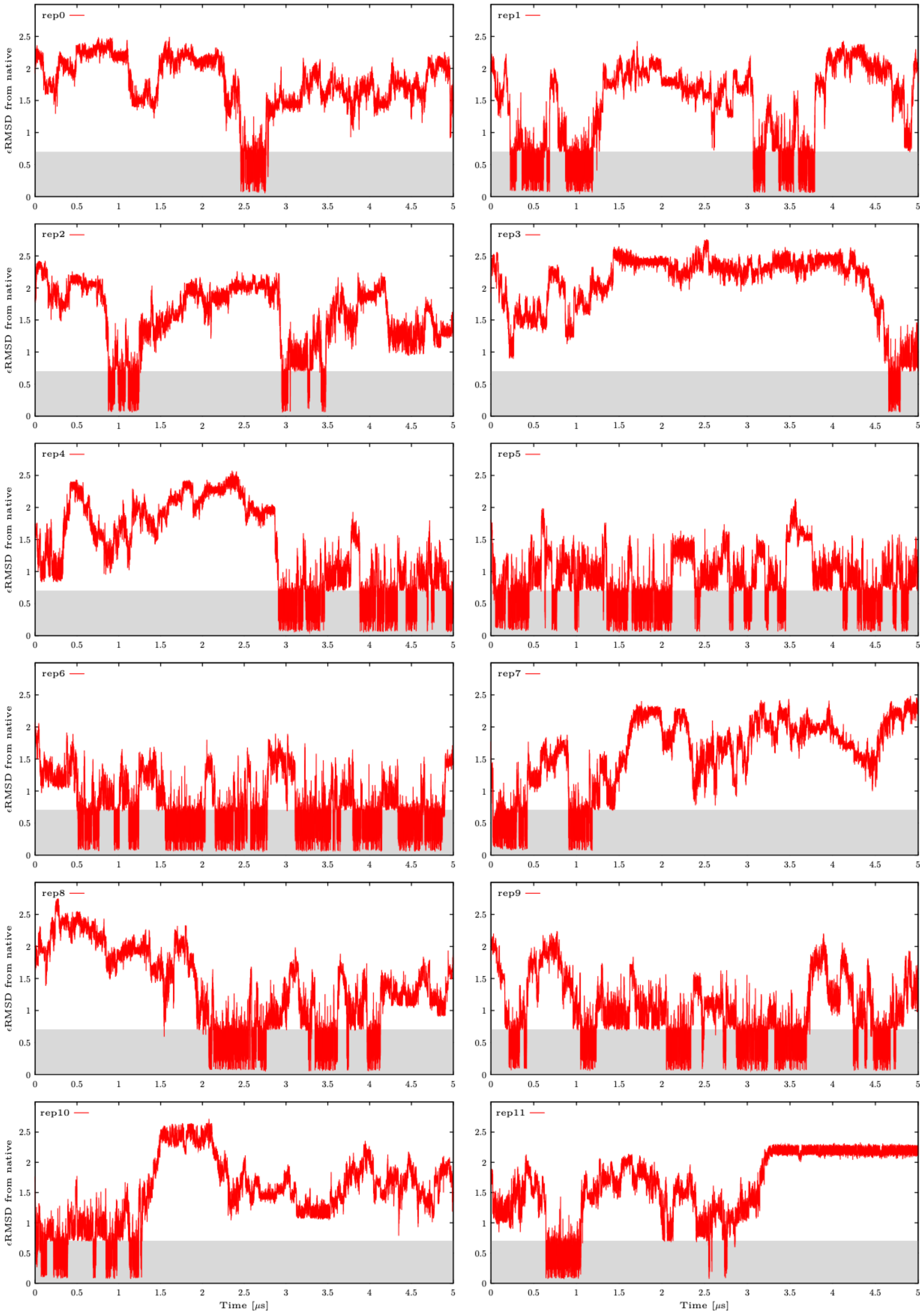

Figure S9: Calculated εRMSD relative to the native state for all twelve continuous (demultiplexed) replicas from the fourth ST-MetaD simulation (5 μs-long) of the 8-mer GAGA TL. See Figure S6 for more details.

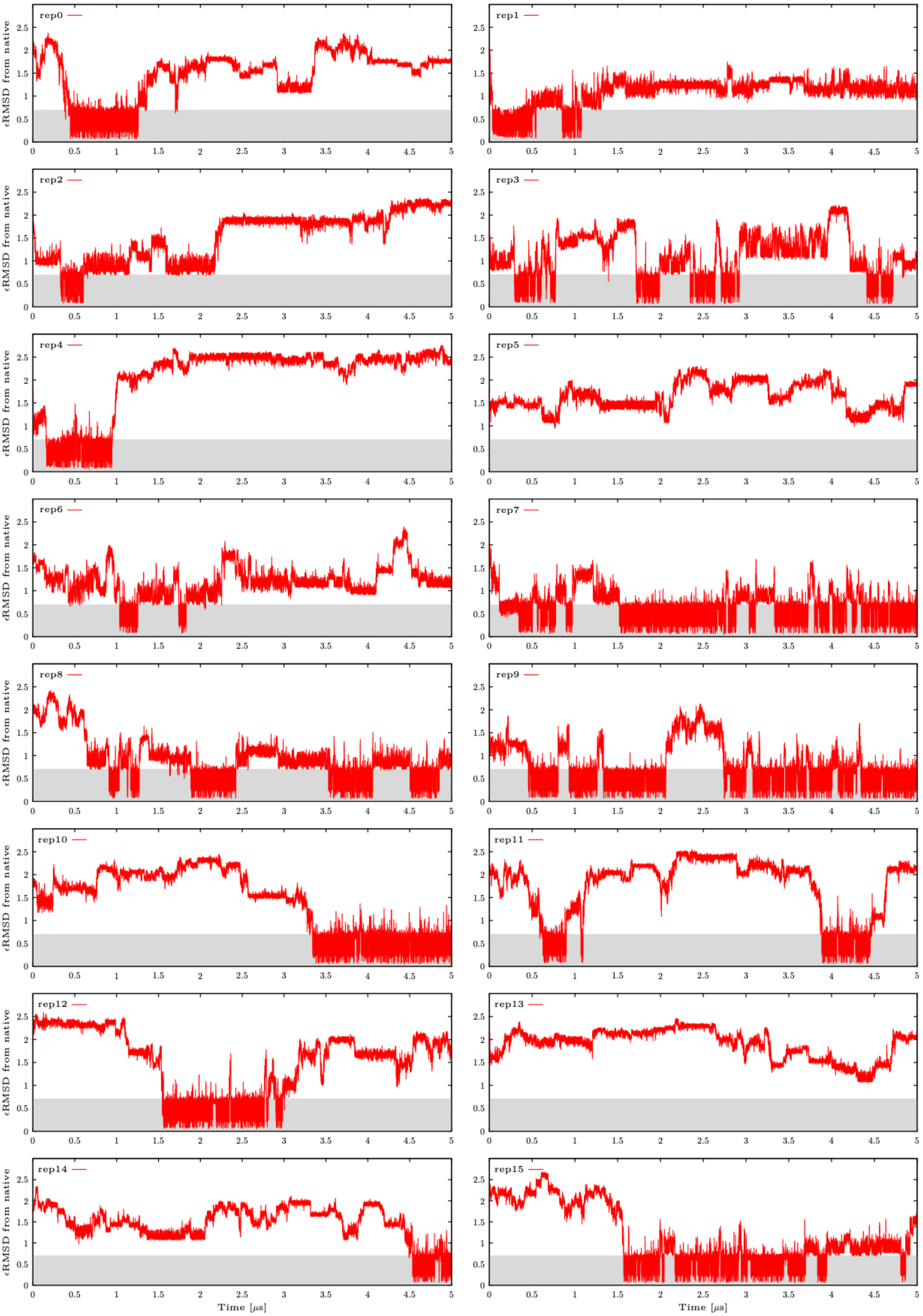

Figure S10: Calculated εRMSD relative to the native state for all sixteen continuous (demultiplexed) replicas from the fifth ST-MetaD simulation (5 μs-long) of the 8-mer GAGA TL. See Figure S6 for more details.

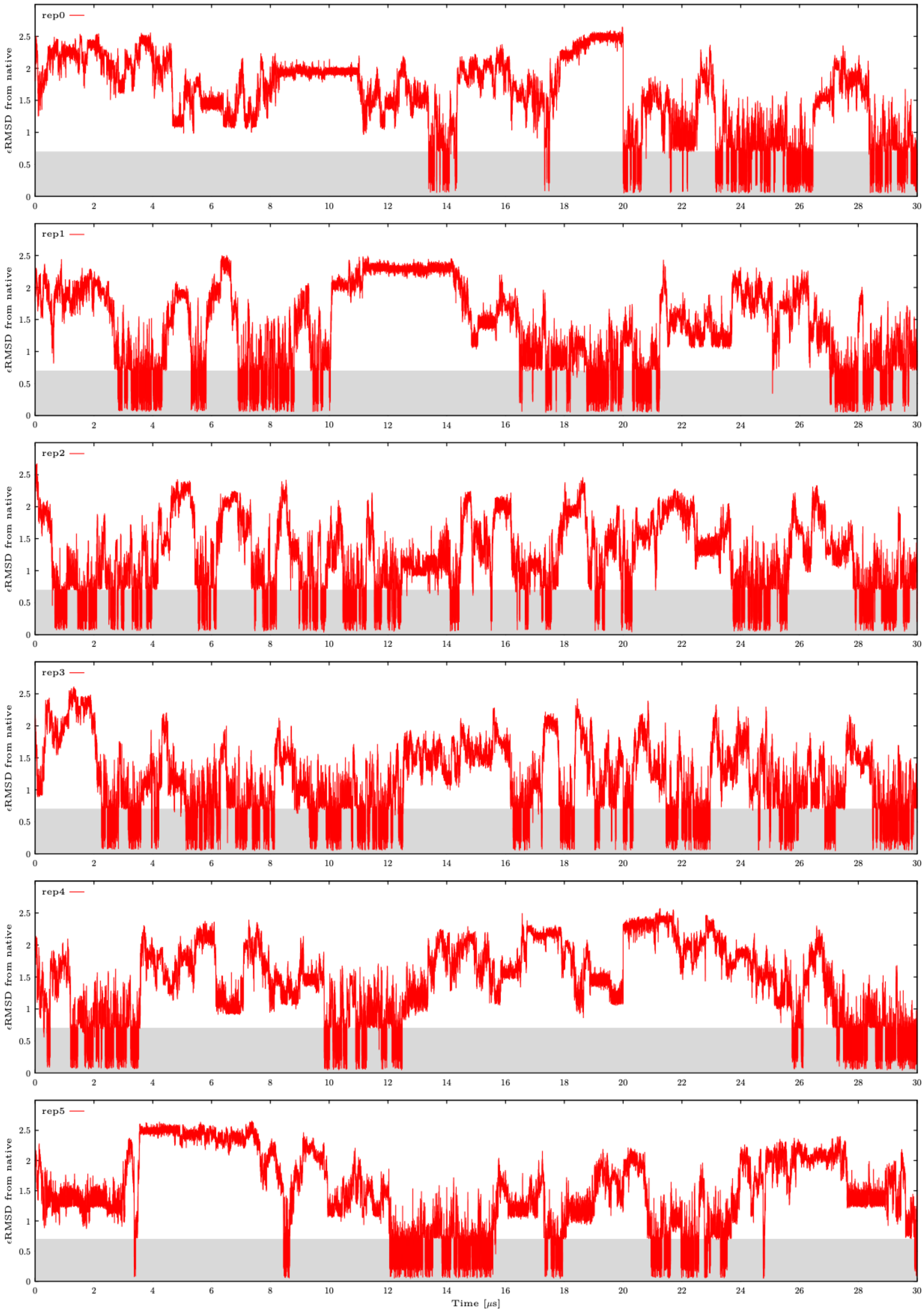

Figure continuing on the next page

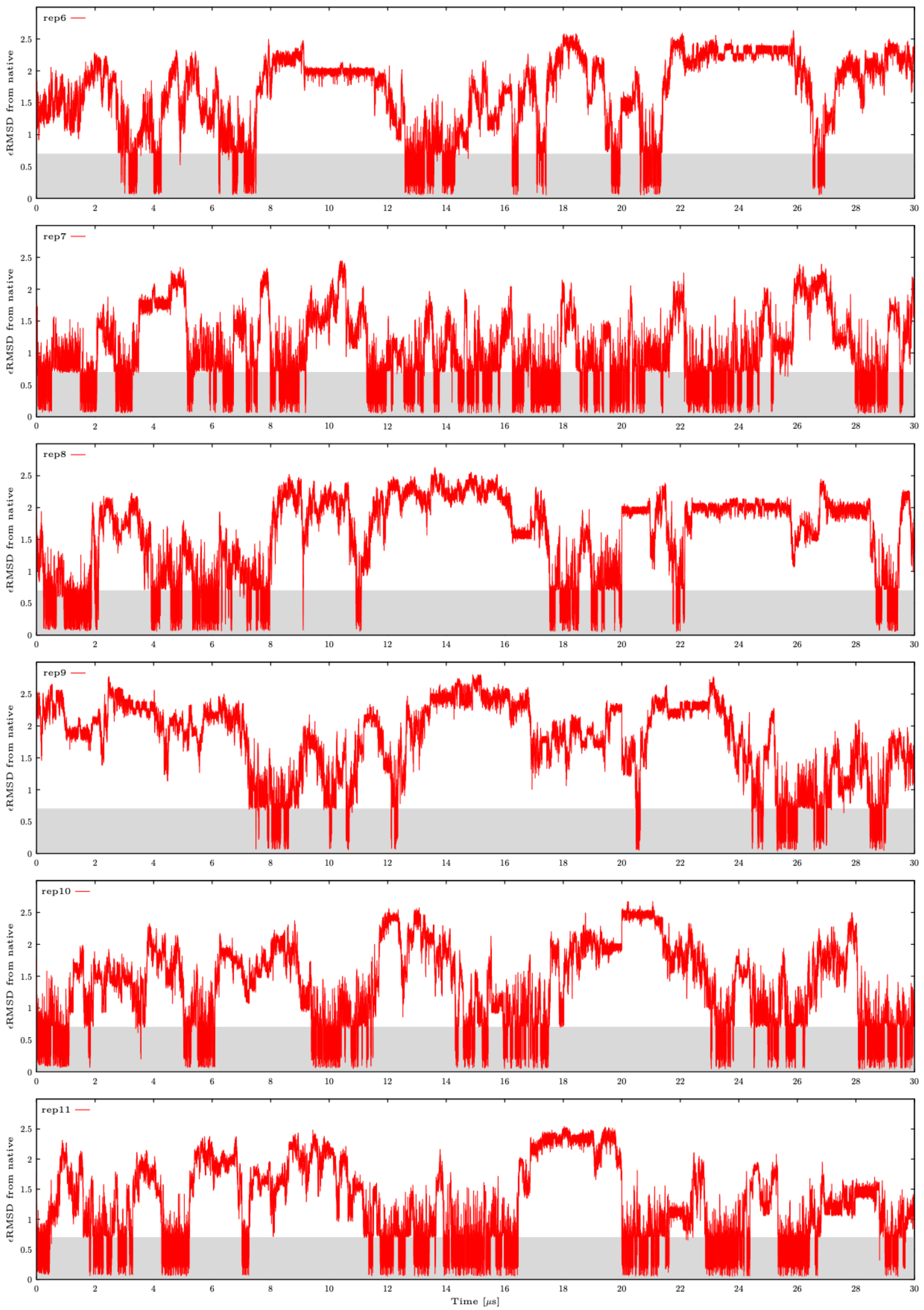

Figure S11: Calculated εRMSD relative to the native state for all twelve continuous (demultiplexed) replicas from the sixth ST-MetaD simulation (30 µs-long) of the 8-mer GAGA TL. See Figure S6 for more details.

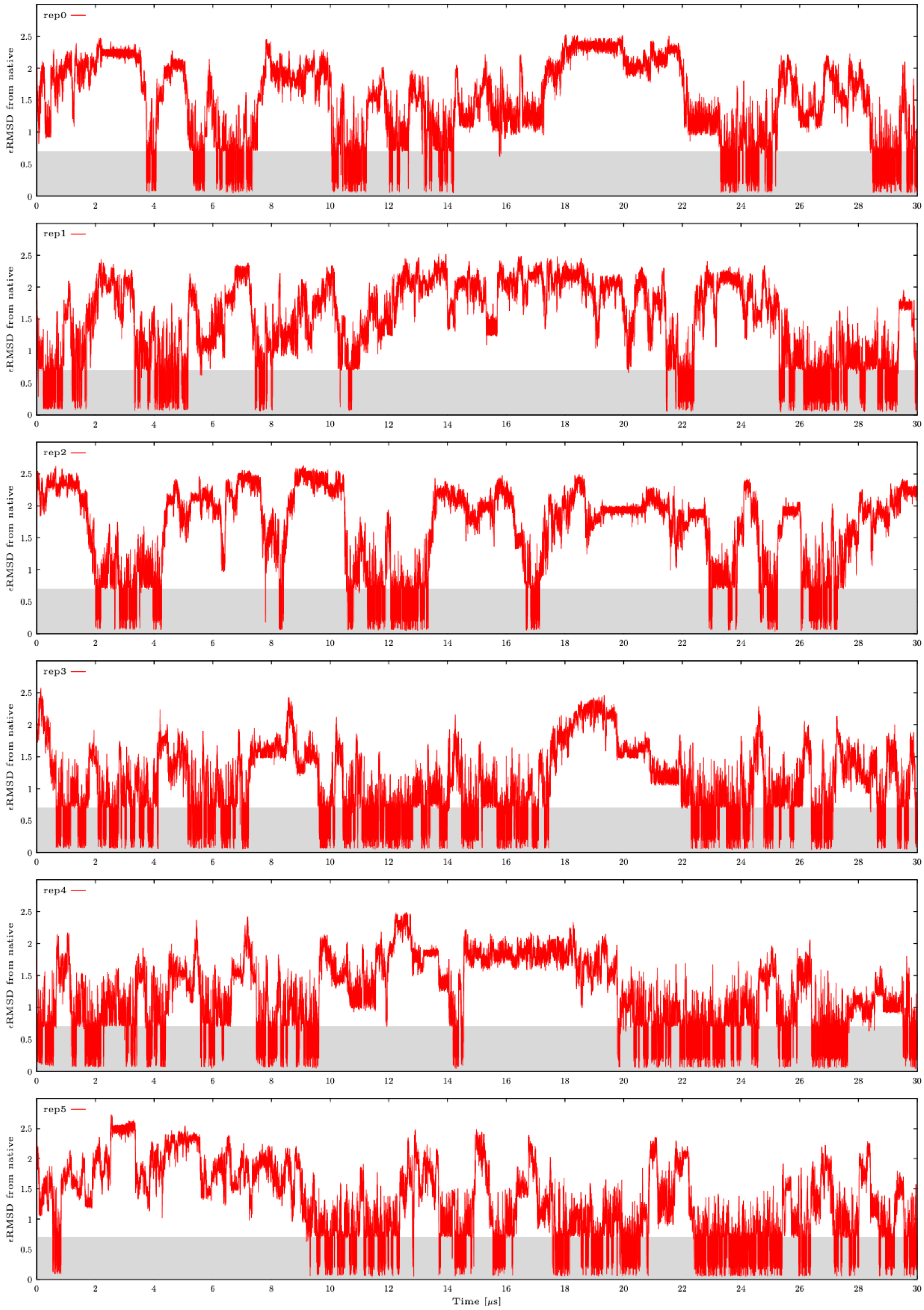

Figure continuing on the next page

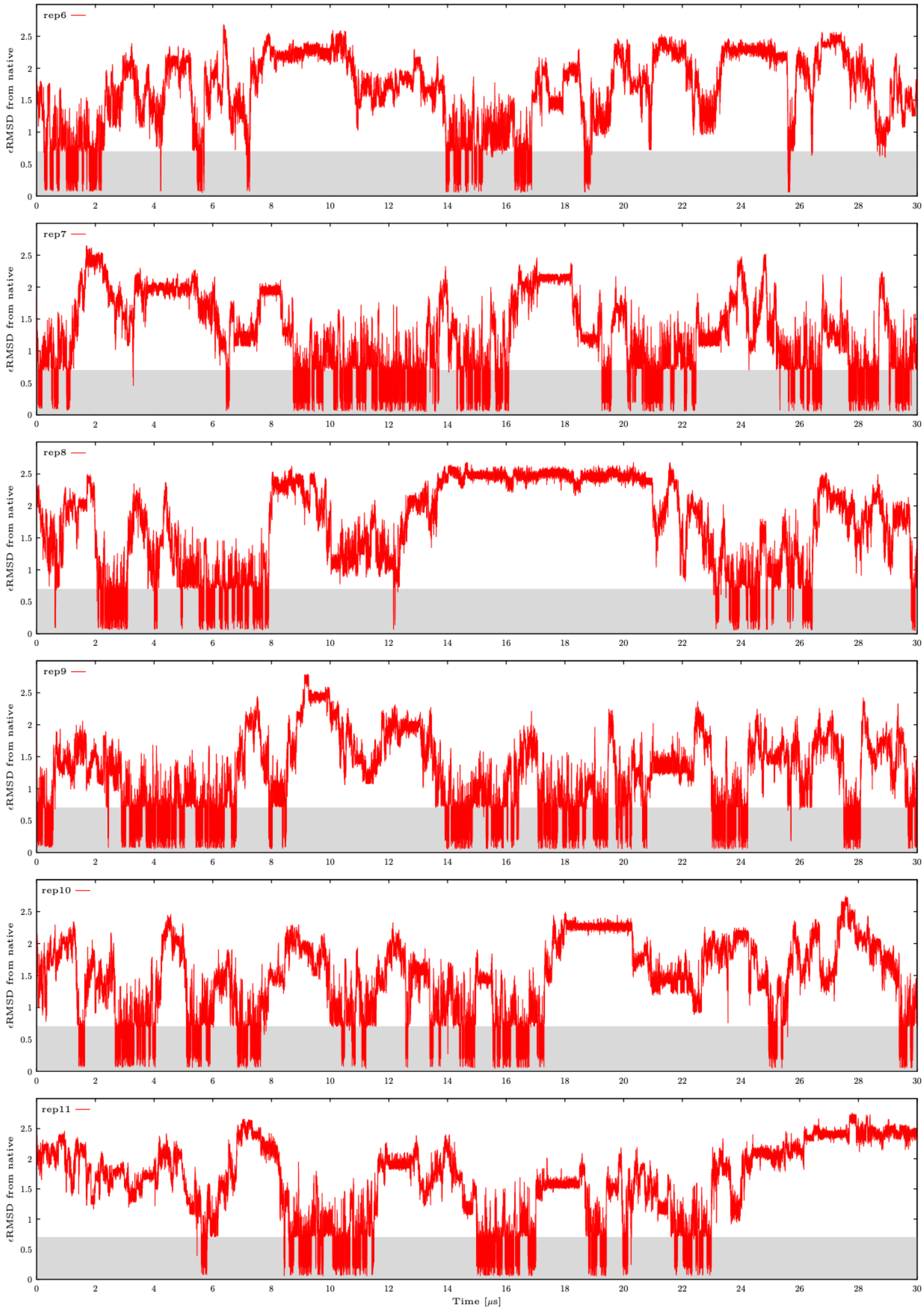

Figure S12: Calculated εRMSD relative to the native state for all twelve continuous (demultiplexed) replicas from the seventh ST-MetaD simulation (30 µs-long) of the 8-mer GAGA TL. See Figure S6 for more details.

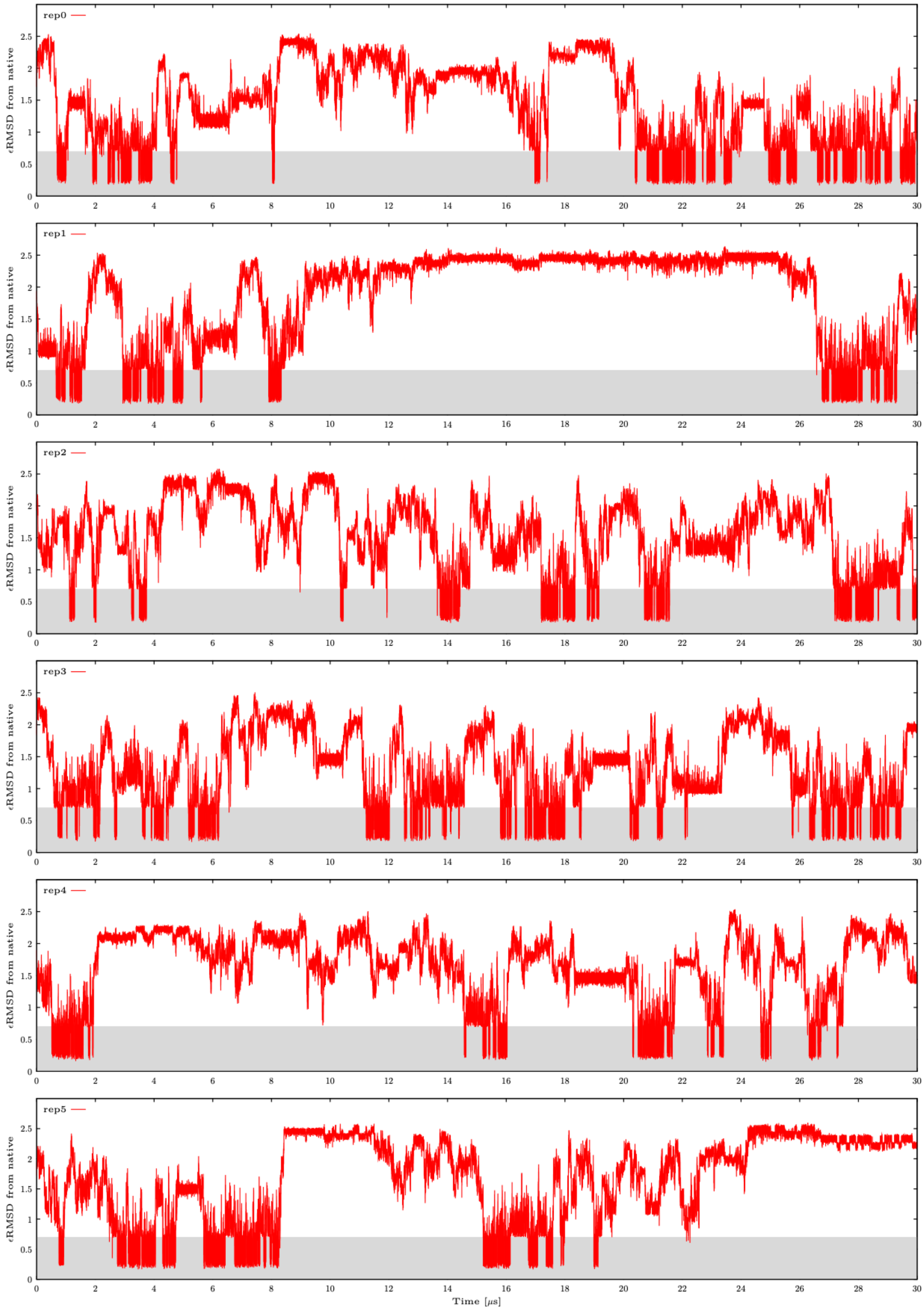

Figure continuing on the next page

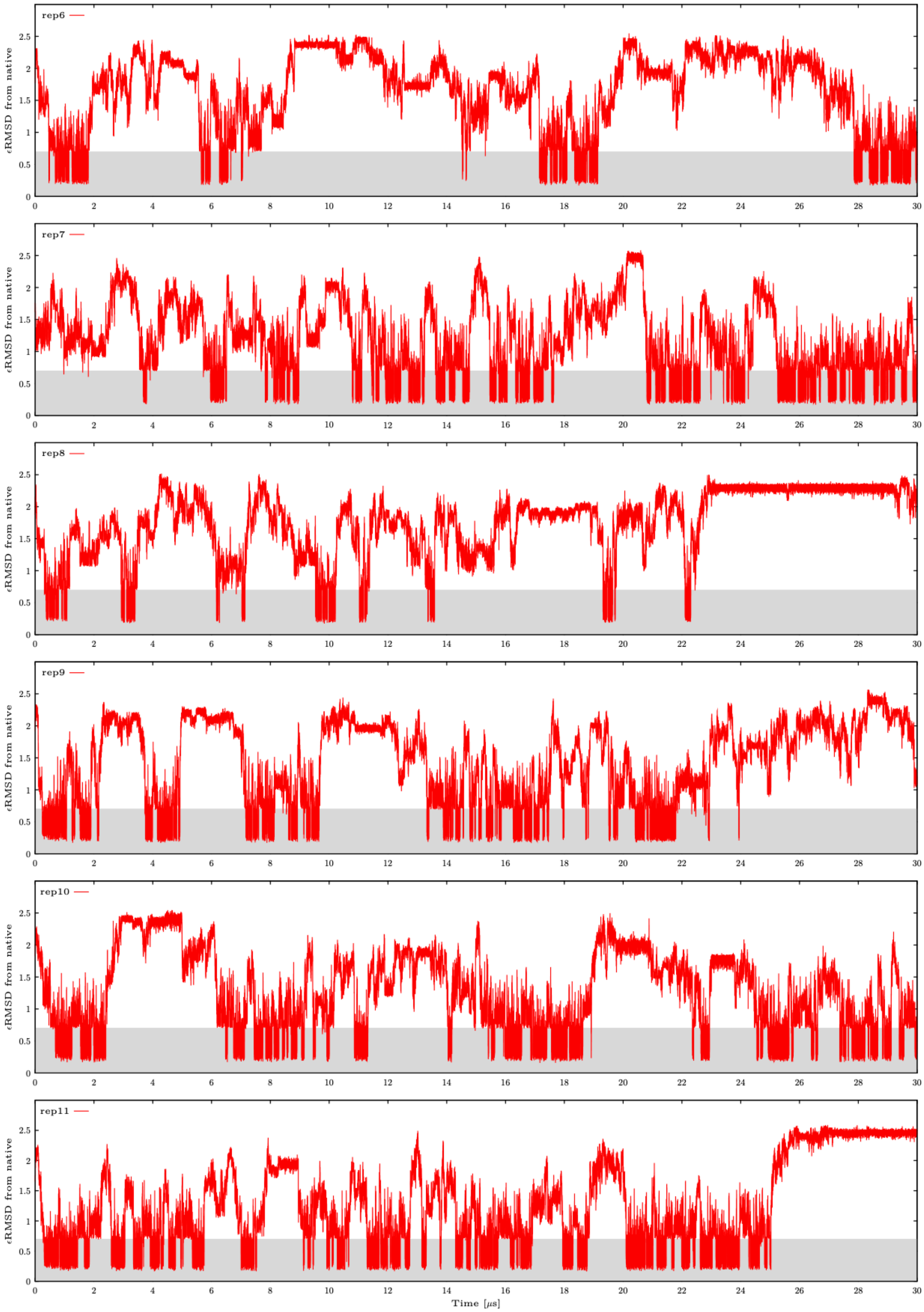

Figure S13: Calculated εRMSD relative to the native state for all twelve continuous (demultiplexed) replicas from the ST-OPES simulation (30 μs-long) of the 8-mer GAGA TL. See Figure S6 for more details.

REFERENCES

(1) Hirao, I.; Nishimura, Y.; Tagawa, Y.; Watanabe, K.; Miura, K. Extraordinarily Stable Mini-Hairpins - Electrophoretical and Thermal-Properties of the Various Sequence Variants of D(Gcgaaagc) and Their Effect on DNA Sequencing. *Nucleic Acids Res* **1992**, *20* (15), 3891-3896. DOI: DOI 10.1093/nar/20.15.3891.

(2) Leulliot, N.; Baumruk, V.; Gouyette, C.; Huynh-Dinh, T.; Turpin, P. Y.; Ghomi, M. Aqueous phase structural features of GNRA tetraloops formed in short hairpins as evidenced by UV absorption and Raman spectroscopy. *Vib Spectrosc* **1999**, *19* (2), 335-340. DOI: Doi 10.1016/S0924-2031(98)00071-X.

(3) Rodriguez, A.; Laio, A. Clustering by Fast Search and Find of Density Peaks. *Science* **2014**, *344* (6191), 1492-1496. DOI: 10.1126/science.1242072.

(4) Bottaro, S.; Di Palma, F.; Bussi, G. The role of nucleobase interactions in RNA structure and dynamics. *Nucleic Acids Res* **2014**, *42* (21), 13306-13314. DOI: 10.1093/nar/gku972.

(5) Mlynsky, V.; Kuhrova, P.; Kuhr, T.; Otyepka, M.; Bussi, G.; Banas, P.; Sponer, J. Fine-Tuning of the AMBER RNA Force Field with a New Term Adjusting Interactions of Terminal Nucleotides. *J Chem Theory Comput* **2020**, *16* (6), 3936-3946. DOI: 10.1021/acs.jctc.0c00228.

(6) Bottaro, S.; Bussi, G.; Pinamonti, G.; Reisser, S.; Boomsma, W.; Lindorff-Larsen, K. Barnaba: software for analysis of nucleic acid structures and trajectories. *RNA* **2019**, *25* (2), 219-231. DOI: 10.1261/rna.067678.118.

(7) Zirbel, C. L.; Sponer, J. E.; Sponer, J.; Stombaugh, J.; Leontis, N. B. Classification and energetics of the base-phosphate interactions in RNA. *Nucleic Acids Res* **2009**, *37* (15), 4898-4918. DOI: 10.1093/nar/gkp468.

(8) Leontis, N. B.; Westhof, E. Geometric Nomenclature and Classification of RNA Base Pairs. *RNA* **2001**, *7* (4), 499-512. DOI: 10.1017/s1355838201002515.
